# Inadvertent Intracranial Nasogastric Tube Placement Causing Traumatic Brain Injury in a Neonatal Cat

**DOI:** 10.1111/jvim.70200

**Published:** 2025-08-31

**Authors:** Riley Shugg, Steven W. Frederick, Sarah A. Moore, Lisa L. Powell

**Affiliations:** ^1^ BluePearl Pet Hospital Tampa Florida USA; ^2^ BluePearl Science Tampa Florida USA; ^3^ BluePearl Pet Hospital Golden Valley Minnesota USA

**Keywords:** brain hemorrhage, complication, cribriform plate, feline, pneumonia, rhinorrhea

## Abstract

A 20‐day‐old, 0.26 kg intact female domestic shorthair kitten was presented for evaluation of labored breathing and decreased appetite. Physical examination and thoracic radiographs were consistent with bronchointerstitial pneumonia, and the cat was hospitalized and treated with antibiotics, oxygen therapy, and nutritional support through a nasogastric tube. Mild resistance was encountered during nasogastric tube placement before advancement to the premeasured length. Lateral thoracic radiographic examination of the thorax, neck, and head suggested the nasogastric tube had entered the calvarium through the cribriform plate and had become coiled. The nasogastric tube was immediately removed, with no acute decline in the kitten's neurologic status; however, the kitten ultimately died secondary to suspected respiratory failure. Postmortem magnetic resonance imaging and necropsy confirmed the presence of severe pneumonia and marked cerebral and midbrain hemorrhage secondary to traumatic intracranial nasogastric tube placement.

## 1 Introduction

Early nutritional support improves outcomes in veterinary medicine [1, 2]. When voluntary feeding is not possible, nutrition might be provided through nasoenteral routes via nasogastric or nasoesophageal intubation. However, complications associated with nasoenteral tube placement are as high as 25.8% in veterinary patients [3].

Nasoenteral intubation into the trachea rather than the esophagus is a potentially devastating complication associated with this technique [4]. To prevent inadvertent administration of nutrition into the respiratory tract, it is common practice to evaluate the placement of the nasoenteral tube in the esophagus or stomach with orthogonal radiography before securing the tube at the nostril [5]. Although this practice effectively prevents the administration of nutritional products into the respiratory system, it does not address other potential complications associated with tube misplacement.

In people, placement of nasoenteral tubes through traumatic palatal defect, defects in the cribriform plate, or a combination of palatal and cribriform plate defects are rarely reported with devastating results including patient death [6, 7, 8]. This case report documents the inadvertent placement of a nasogastric tube through the cribriform plate and into the calvarium and brain parenchyma in a kitten, a complication not reported in veterinary medicine.

## 2 Case Description

A 20‐day‐old, 0.26 kg intact female domestic shorthair kitten was presented to the emergency department of a veterinary specialty hospital for open‐mouth breathing, decreased nursing vigor, and inability to maintain or gain weight. The queen, who was housed with the kitten, was previously diagnosed with an upper respiratory infection. On presentation, the kitten's temperature was normal for a neonate at 99.6°F (37.6°C), heart rate was 200 beats/min, and respiratory rate was 40 breaths/min. Physical examination abnormalities included a quiet mentation, intermittent open‐mouth breathing, tachypnea with labored breathing and increased lung sounds, and a normal hard palate. The neurologic examination at that time, although limited, documented normal age‐appropriate ambulation and a cranial nerve examination that was considered normal for the age of the animal.

At presentation, blood glucose was 171 mg/dL (reference interval 80–120 mg/dL). Thoracic and abdominal radiographs were performed and showed right middle lung lobe consolidation with pneumonia as the primary differential, widespread bronchointerstitial lung pattern attributed to bronchitis, a distended stomach suspected secondary to aerophagia, and decreased abdominal detail considered normal for age and body condition.

**FIGURE 1:**
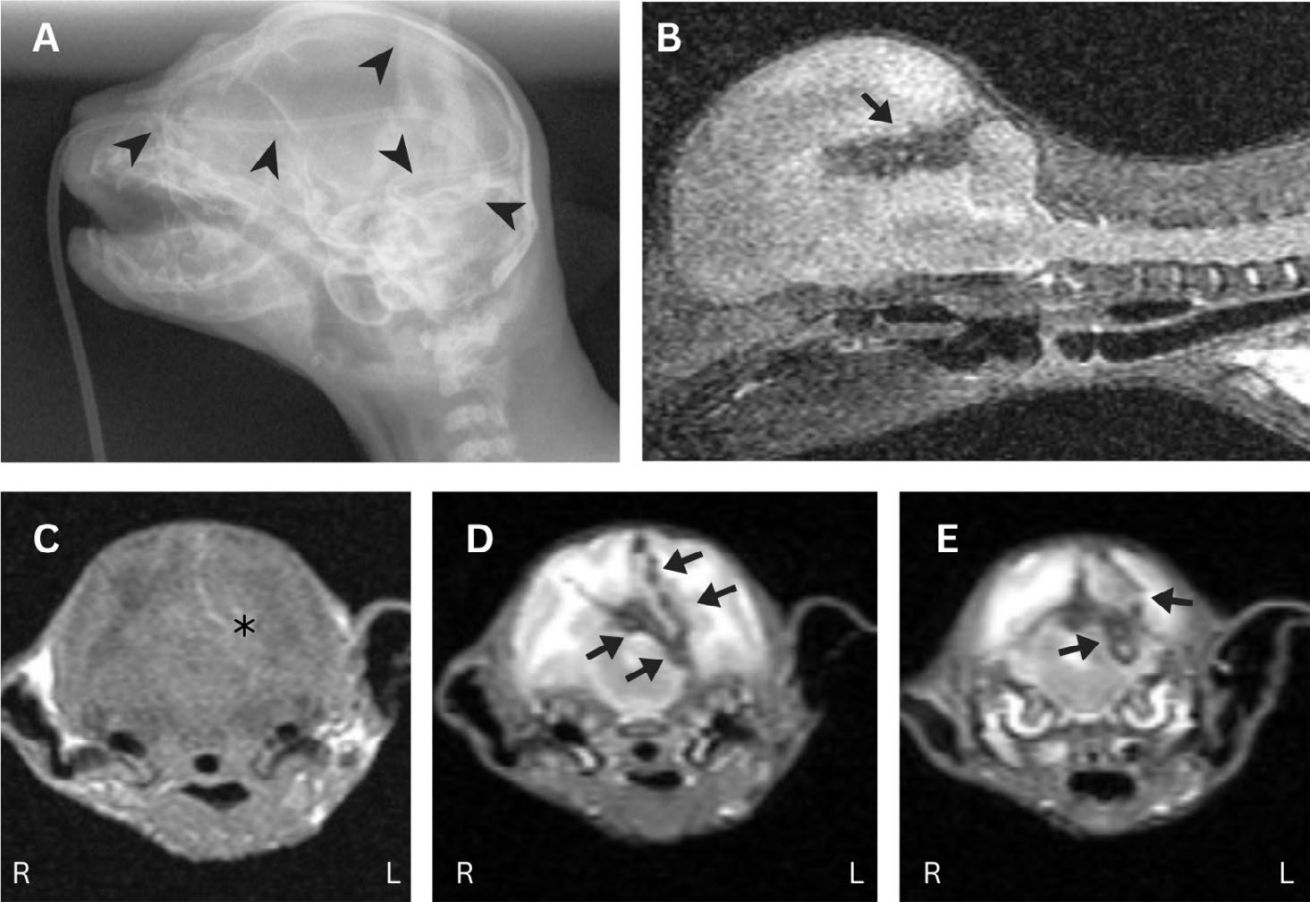
Radiograph (A) and postmortem magnetic resonance images (MRI; B–E) of a 3‐week‐old domestic shorthair kitten with inadvertent intracranial nasogastric tube placement. The nasogastric tube entered the calvarium through the cribriform plate, coiled within the caudal fossa and redirected back through the cranial fossa (A; arrowheads). T2‐weighted midline sagittal (B) and T1‐weighted (C) and T2‐weighted (D and E) transverse images acquired at the level of the tympanic bullae and the cerebellar tentorium revealed hemorrhagic tracts extending through the right cerebral hemisphere (C; asterisk), crossing midline to involve the left cerebral hemisphere (B, D; arrows) and cerebellar tentorium and caudal fossa (E; arrows).

An intravenous catheter was attempted but could not be placed; a needle intraosseous catheter was not attempted due to the ability to utilize subcutaneous fluid administration and overall satisfactory patient stability at that time. After initial thoracic radiographs and radiographic confirmation of suspected pneumonia, the kitten was prescribed ampicillin‐sulbactam (30 mg/kg SC q 8 h), and supplemental oxygen support at 40% in an oxygen cage. Since neonates have increased (120–180 mL/kg/day) hydration needs compared to adults, subcutaneous fluid administration (58 mL/kg [15 mL] Plasmalyte q 12 h) was performed to treat moderate dehydration and to maintain an adequate hydration status [9]. Due to inappetence and unwillingness to accept enteral nutrition from a bottle, a 3.5 French red rubber catheter was placed as a nasogastric tube, and appropriate placement in the esophagus and stomach was verified with radiographic examination before administration of kitten milk replacer (5 mL every 4 to 6 h, KMR, PetAg, Hampshire, IL) through the tube. However, the kitten removed the nasogastric tube 6 h later, and it was not initially replaced. Attempts to bottle feed were continued, and oral dextrose (2 mL/kg 50% dextrose, diluted 1:1 with saline PO q 1 h) was prescribed alongside intermittent bottle feeding. Twenty‐four hours after hospitalization, a veterinary criticalist attempted to replace the 3.5 French red rubber nasogastric tube. Some difficulty was initially encountered while passing the tube through the nasal cavity, but passage was eventually successful. A lateral thoracic radiograph was performed to confirm tube placement, but the tube was absent from the esophagus and stomach. A second lateral radiograph was performed to include the kitten's head, neck, and thorax, and the nasogastric tube was noted to be coiled within the head, and there was strong suspicion that it was located within the calvarium, although this was not confirmed with an orthogonal view (Figure 1A). Radiographic interpretation by a board‐certified radiologist was consistent with passage of the nasogastric tube through the cribriform plate and into the calvarium, although an orthogonal view was required for confirmation of tube location. Immediately after removal of the nasogastric tube, the kitten had progressive dyspnea and was subjectively less responsive; an IV catheter placement was again attempted but was unsuccessful. Empiric oral dextrose was administered (4 mL/kg 50% dextrose), and the kitten's mentation improved and remained quiet but responsive throughout the night. Due to lack of nasogastric tube and persistent patient inappetence, intermittent orogastric feedings were implemented with a 5 French red rubber catheter (5 mL KMR, q 4 h) along with oral dextrose (2 mL/kg 50% dextrose, diluted 1:1 with saline PO q 1 h).

After the removal of the nasogastric tube, fluid was noted from the nares and in the back of the mouth, and a bulb syringe was used to evacuate the fluid from both sites every 6 h with no noticeable decrease in fluid production during hospitalization. On the 3rd day of hospitalization, the kitten remained quiet but responsive and was noted to have decreased fluid production from the nares and in the back of the mouth. At this time, the kitten was ambulatory with generalized weakness and was occasionally falling to the right side. There was a persistent mildly increased respiratory effort present in room air that was not improved with 40% oxygen. Continued hospitalization was recommended, but discharge was elected by the owner, and the kitten was discharged with oral amoxicillin‐clavulanic acid (25 mg/kg, PO q 12 h) and instructions to suction fluid from the nares and back of the mouth every 6 h.

The kitten was again presented to the emergency department for increased respiratory effort and open mouth breathing 8 h later. One dose of amoxicillin‐clavulanic acid and one bottle feeding had been administered at home that evening. An abbreviated neurologic examination was performed at that time and revealed a quiet but responsive mentation and no other overt changes to neurologic status. The kitten remained ambulatory with persistent falling to the right and had persistently increased respiratory rate and effort, both of which had been present at the time of previous discharge from the hospital. Hospitalization was elected, and treatment included administration of 40% oxygen, resumption of subcutaneous fluid therapy due to continued inability to achieve intravenous catheterization, amoxicillin‐clavulanic acid, and orogastric tube feeding after the previously prescribed orders.

Due to persistent pneumonia, the amoxicillin‐clavulanic acid was discontinued, sulfamethoxazole/trimethoprim (0.4 mg/kg PO q 12 h) was prescribed to provide a broader spectrum of coverage, and saline nebulization therapy was added. Neurologic examination at this time revealed a dull mentation, which was thought to be due to the kitten's systemic disease, related to previous traumatic nasogastric tube placement, or a combination of the two factors. Throughout the day, the kitten developed progressive tachypnea with persistent dyspnea. Neurologic deterioration was not noted during this time, but the ability to complete a full neurologic assessment was hampered by progressive respiratory distress. On that day, 5 days after the initial presentation, the kitten arrested due to suspected respiratory failure, and resuscitation was not attempted per the owner's request.

A postmortem magnetic resonance imaging study of the kitten's brain was performed. Findings were consistent with traumatic introduction and subsequent removal of a foreign object, including hemorrhagic tracts extending through the right cerebral hemisphere, crossing midline to involve the left cerebral hemisphere and cerebellar tentorium (Figure 1B–E).

A necropsy was performed by a board‐certified veterinary pathologist. Evaluation of the nervous system showed blood coagulum within the longitudinal fissure and supratentorial aspect of the tentorium cerebelli. The occipital region of the left cerebral hemisphere was discolored dark red in a poorly demarcated region. Histopathology of the brain showed locally extensive hemorrhage bridging both hemispheres but was more severe on the left side. These changes were consistent with multifocal to locally extensive moderate to marked cerebrum to midbrain hemorrhage, acute to subacute in duration, which was worse on the left and likely related to the reported inadvertent intracranial placement of a nasogastric tube. Evaluation of the respiratory system was consistent with bronchointerstitial pneumonia with viral inclusion bodies, likely secondary to feline herpes virus. Aerobic and anaerobic bacterial culture of the lung tissue showed no growth.

## 3 Discussion

Intracranial placement of nasogastric tubes has not been reported in veterinary medicine, though complications associated with nasogastric tube placement are commonly reported [3, 4, 5]. Intracranial placement of nasogastric tube is a rare but known complication in people, typically seen after head trauma and cribriform plate fracture.

Documented consequences of inadvertent intracranial nasogastric tube placement in people include neurological deficits such as hemiparesis, vision loss, cerebral hemorrhage, and brain death, with an overall reported mortality rate of 64% [7]. After intracranial tube placement, the kitten in this case report had generalized weakness with occasional episodes of falling to the right. While a complete neurologic examination was not documented, and neurologic examinations on neonates can be inherently challenging, the signs are attributed to hemiparesis associated with a left thalamocortical lesion likely concurrent with weakness secondary to overall poor systemic health. Alternatively, a left paradoxical vestibular lesion could have been considered. The neurolocalization to the left thalamocortex is supported by postmortem findings demonstrating multifocal hemorrhagic lesions worse in the left midbrain and left caudal cerebral hemisphere. Histopathologic findings of the brain were similar and documented hemorrhage that was acute to subacute in nature and considered consistent with the previously noted timeline of traumatic tube placement 4 days before death. Although the presumed cause of death in this kitten was respiratory failure due to chronic viral pneumonia, based on necropsy findings in the brain, neurotrauma associated with the nasogastric tube could have been a contributing factor.

Cerebrospinal fluid rhinorrhea is a secondary complication associated with intracranial (trans‐cribriform plate) nasogastric tube placement in people, and in some cases only resolves with surgical closure of the defect [8]. The kitten in this case developed a nasal and postnasal discharge after intracranial nasogastric tube placement, though the fluid was not collected for analysis. In this case, a bulb syringe was used to remove fluid from the nares and the back of the mouth every 6 h. While this might have been cerebrospinal fluid based on the timing of the development of the discharge, other considerations include secretions related to the concurrent pneumonia or secondary to local irritation from the placement of the tube.

Numerous modalities for evaluating nasogastric tube placement are described in people, dogs, and cats, including negative pressure, capnography tracings, auscultation evaluations, and ultrasound [3, 5, 7]; however, in human and veterinary medicine, radiographic imaging remains the standard of care [3, 5, 7]. In veterinary practice, a single lateral thoracic radiographic image is used to ensure that the nasogastric tube is dorsal to the trachea and the carina, confirming location within the esophagus and not the respiratory tract. That protocol was initially followed in this case until the presence of the tube was noted to be absent from that image, when a second lateral radiograph including the head and neck was performed. A ventrodorsal radiograph of the skull was not performed for confirmation of tube placement within the calvarium; however, postmortem magnetic resonance imaging and postmortem evaluation demonstrated tracts of hemorrhage within the brain consistent with the suspected diagnosis of trauma secondary to inadvertent intracranial nasogastric tube placement.

There are multiple modalities for evaluation of correct placement of nasogastric tubes in both people and veterinary species; however, most are aimed at avoiding inadvertent placement into the respiratory tract. Since the goal of the currently used modalities is focused on prevention of complications occurring after placement, additional evaluation modalities might be warranted for cases with altered anatomy associated with trauma or age (e.g., neonates having small nares prohibiting medial and ventral tube guidance). In these cases, additional radiographic views or continuous fluoroscopic evaluation of the feeding tube entering the nares, nasal cavity, and passing beyond the sinuses before any resistance should be considered. If the cat in this study were to have continued care, additional considerations for treatment might have centered around pain management, minimizing secondary increased intracranial pressure due to hemorrhage, and possible surgical correction of the anatomic defects associated with the nasogastric tube placement.

This case illustrates a rare complication that might occur secondary to nasogastric tube placement in veterinary patients, further demonstrating the importance of proper placement techniques and consideration for modalities such as fluoroscopy to guide appropriate nasogastric tube placement.

## Disclosure

Authors declare no off‐label use of antimicrobials.

## Ethics Statement

Authors declare no institutional animal care and use committee or other approval was needed. Authors declare human ethics approval was not needed.

## Conflicts of Interest

Sarah A. Moore serves as Associate Editor for the Journal of Veterinary Internal Medicine. She was not involved in the review of this manuscript. The other authors declare no conflicts of interest.
